# A novel missense variant in TNFAIP3 associated with autoimmunity reveals the contribution of STAT1/mTOR pathways

**DOI:** 10.1093/cei/uxaf048

**Published:** 2025-07-25

**Authors:** Judith Potjewijd, Hans J P M Koenen, Caspar I van der Made, Esther van Rijssen, Xuehui He, Renee Ysermans, Liset Ungethum, Ruud Theunissen, Leon J Schurgers, Jan Damoiseaux, Pieter van Paassen, Ruben L Smeets

**Affiliations:** Department of Clinical and Experimental Immunology, Maastricht University Medical Center, Maastricht, The Netherlands; Department of Laboratory Medicine, Laboratory of Medical Immunology, Radboud University Medical Center, Nijmegen, the Netherlands; Department of Human Genetics, Radboud University Medical Center and Radboud Institute for Molecular Life Sciences, Nijmegen, the Netherlands; Department of Internal Medicine and Radboud Center for Infectious Diseases (RCI), Radboud University Medical Center and Radboud Institute for Molecular Life Sciences, Nijmegen, the Netherlands; Department of Laboratory Medicine, Laboratory of Medical Immunology, Radboud University Medical Center, Nijmegen, the Netherlands; Department of Laboratory Medicine, Laboratory of Medical Immunology, Radboud University Medical Center, Nijmegen, the Netherlands; Department of Clinical and Experimental Immunology, Maastricht University Medical Center, Maastricht, The Netherlands; Department of Biochemistry, Cardiovascular Research Institute Maastricht, Maastricht University, Maastricht, The Netherlands; Department of Clinical and Experimental Immunology, Maastricht University Medical Center, Maastricht, The Netherlands; Department of Biochemistry, Cardiovascular Research Institute Maastricht, Maastricht University, Maastricht, The Netherlands; Central Diagnostic Laboratory, Maastricht University Medical Center, Maastricht, The Netherlands; Department of Clinical and Experimental Immunology, Maastricht University Medical Center, Maastricht, The Netherlands; Department of Laboratory Medicine, Laboratory of Medical Immunology, Radboud University Medical Center, Nijmegen, the Netherlands; Department of Laboratory Medicine, Radboudumc Laboratory for Diagnostics, Radboud University Medical Center, Nijmegen, The Netherlands

**Keywords:** A20 haploinsufficiency, TNFAIP3, polyautoimmunity, NF-κB, STAT1 and mTOR pathway

## Abstract

Heterozygous loss-of-function mutations in the *TNFAIP3* gene lead to A20 haploinsufficiency (HA20). A20 protein is a negative feedback regulator of NF-κB signaling. Traditionally, HA20 is associated with Behçet's disease-like symptoms, however, recent findings suggest it may also manifest with a broader array of autoimmune diseases. Here, we describe a novel *TNFAIP3* variant in a Dutch family, predominantly presenting with polyautoimmunity rather than autoinflammatory manifestations. We evaluated two patients from a Dutch family with autoimmune symptoms. Whole-exome sequencing (WES) identified a heterozygous c.608T>G (p.Leu203Arg) missense variant in *TNFAIP3*, located within the OTU domain. Functional analyses included immunoblotting of peripheral blood mononuclear cells (PBMCs) and an overexpression model using transfected HEK293T cells. A20 protein expression was evaluated, while phosphoflow cytometry assessed phosphorylation of key signaling molecules in the NF-κB, STAT and mTOR pathways. Inflammatory cytokine production was measured in cell culture supernatants. Overexpression of this missense A20 variant in HEK293T enhanced NF-κB signaling, reflected by increased TRAF6 expression and IκBα phosphorylation. Patient-derived PBMCs demonstrated reduced A20 expression, increased phosphorylation within the NF-κB, STAT1, and mTOR pathways, and elevated production of pro-inflammatory cytokines. These molecular alterations suggest disrupted immune regulation contributing to the observed autoimmune phenotype. The identification of this novel *TNFAIP3* variant contributing to HA20 expands the clinical spectrum to include predominant autoimmune manifestations. In addition to NF-κB and STAT1 activation, we discovered mTOR pathway activation, shedding new light on A20’s function and progression toward autoimmunity. Furthermore, the involvement of mTOR pathway also provides new therapeutic possibilities.

## Teaser

This study identifies a novel TNFAIP3 variant in a Dutch family, showing that HA20 can primarily present with autoimmune symptoms rather than just Behçet's disease-like traits. Functional analysis of patient cells revealed decreased A20 expression and increased activation of NF-κB, STAT1, and mTOR pathways, which contribute to heightened immune responses. This expands the clinical understanding of HA20 and suggests potential therapeutic avenues targeting the mTOR pathway for HA20-related autoimmune conditions.

## Graphical Abstract

**Graphical Abstract:**
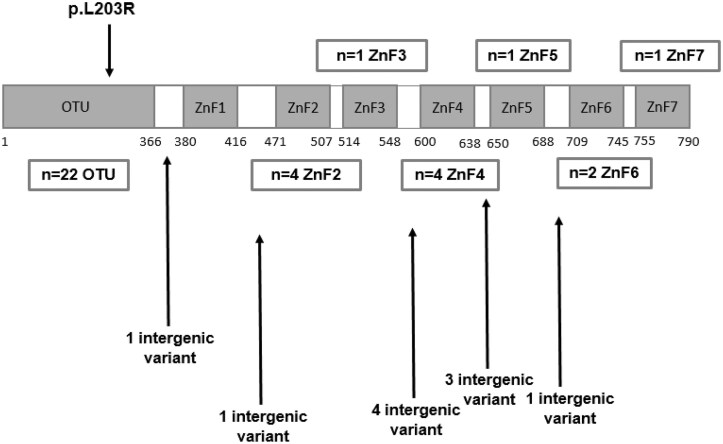

## Introduction

Heterozygous loss-of-function mutations in the *tumor necrosis factor α (TNFα)-induced protein* 3 (*TNFAIP3*) gene, resulting in haploinsufficiency of the A20 protein (HA20), are associated with an early-onset autoinflammatory disease. A20, encoded by *TNFAIP3*, consists of an amino-terminal OTU (ovarian tumor) domain with deubiquitinase activity and seven carboxyl-terminal zinc finger domains with ubiquitin ligase and binding activities. It acts as a potent inhibitor of the pro-inflammatory nuclear factor-κB (NF-κB) signaling pathway, pivotal in immune cell activation. HA20 was initially presented as a condition resembling Behcet‘s disease (BD), characterized by recurrent oral and genital ulcers, fever, arthralgia/arthritis, and gastrointestinal symptoms [1]. Subsequently, there were reports of patients with antibody deficiency and the presence of autoimmune diseases, including autoimmune thyroiditis, systemic lupus erythematosus (SLE) and type 1 diabetes [2–7]. A predominant autoimmune phenotype has been observed in one-third of recently reviewed HA20 patients [8], although the differences in the disrupted inflammatory pathways between these patients and those with more autoinflammatory presentations have not yet been fully elucidated.

Besides the observed decrease in inhibition of the NF-κB signaling pathway in HA20 patients, hyperactivation of the IFNγ signaling pathway has also been noted [5, 9, 10]. Although this supports a rationale for the therapeutic use of JAK1/2 inhibitors, the precise role in the development of autoimmunity has not yet been completely understood in HA20 patients.

Here, we present a novel *TNFAIP3* gene variant (c.608T > G [p.Leu203Arg]) observed in a Dutch family, leading to a predominantly autoimmune phenotype. We conducted an extensive functional evaluation to establish the pathogenicity of this newly identified variant.

## Methods and materials

### Patients

Patient 1, the mother, initially presented at 49 years old at the Maastricht University Medical Center with acute lower extremity ischemia. The CT angiography revealed an occlusion of the common iliac artery, without features typical of vasculitis. This condition developed following a bout of gastroenteritis accompanied by high fever. Her medical history included a childhood history of oral and genital ulcers. At the age of 18, she was diagnosed with erosive rheumatoid arthritis, positive for rheumatoid factor (RF). Later in life, she received diagnoses of pernicious anemia, Hashimoto’s disease, IgA deficiency, and insulin-dependent type 1 diabetes mellitus.

The arterial occlusion of the common iliac artery resulted in the complete absence of arterial blood flow in the lower leg. Despite undergoing a percutaneous transluminal angioplasty, she ultimately required a lower leg amputation. Post-surgery, purpura persisted on both legs. A skin biopsy revealed leucocytoclastic vasculitis in small and medium-sized vessels, accompanied by thrombosis. Serological investigations revealed an elevated serum C-reactive protein (CRP) level (147 mg/L), leukopenia due to low neutrophil counts, positive IgM RF but normal C3 and C4 levels, and the presence of anti-beta2-glycoprotein (GP) I IgG antibodies (26 U/mL, reference value >10 U/mL is considered positive). Additionally, positive anti-thyroid peroxidase (TPO) and anti-parietal cell antibodies supported the prior diagnoses of Hashimoto’s disease and pernicious anemia. Positive anti-glutamic acid decarboxylase suggested an autoimmune etiology for her diabetes. Determining lupus anticoagulant was unreliable due to ongoing treatment with low-molecular-weight heparin. Consequently, the patient received a diagnosis of antiphospholipid syndrome (APS) with both thrombosis and vasculitis. A cardiac source of embolism had already been excluded. Anticoagulant treatment was continued, and she initially received treatment with methylprednisolone and oral cyclophosphamide at a dosage of 2 mg/kg body weight, which resulted in the resolution of inflammation, purpura, and leukopenia. Subsequently, she transitioned to rituximab treatment (2 × 1000 mg intravenously), achieving sustained remission.

Patient 2, the daughter, presented at 29 years old with extensive hematomas due to severe thrombocytopenia (10 × 10E9/L), diagnosed as immune-mediated thrombocytopenia (ITP). She had experienced recurring oral and genital ulcers since adolescence. Her medical history included frequent upper respiratory tract infections, for which she had undergone endoscopic sinus surgery. Examination revealed histologically confirmed sterile neutrophilic folliculitis and symmetric polyarthritis affecting the proximal interphalangeal joints. Laboratory investigations indicated a mildly elevated CRP level (13–17 mg/L) and an IgG2 subclass deficiency (IgG2 0.19 g/L; reference range 1.50–6.40 g/L). Anti-GAD65 antibodies were positive, although there were no signs of insulin deficiency. Initially, she received high doses of oral dexamethasone for ITP, leading to platelet count normalization. Subsequently, she underwent treatment with infliximab, which resulted in the complete resolution of her symptoms. To manage her recurrent upper respiratory tract infections, she received azithromycin as maintenance treatment. Within a year, however, she developed recurrent symptoms including arthritis, diarrhea, and recurrent sinusitis, for which she was initially treated with adalimumab, but due to insufficient response, she was ultimately also treated with rituximab. Additionally, an immunoglobulin replacement therapy was initiated.

Informed consent for *in silico* whole-exome sequencing (WES) gene panel analysis for inborn errors of immunodeficiency (IEI) and subsequent functional assays was obtained from both patients. Explicit written consent was given for publication of research findings.

### Diagnostic whole-exome sequencing

WES was performed as described previously [11]. In brief, whole-blood genomic DNA extracted from the mother was processed at the Beijing Genomics Institute (BGI) Europe (BGI Europe, Copenhagen, Denmark). Exonic DNA was enriched using the Agilent (Agilent Technologies, Santa Clara, CA) exome kit and sequenced on Illumina HiSeq4000 (Illumina Sequencing, San Diego, CA) with 2 × 150 base pair paired-end sequencing. Downstream processing was performed using an automated data analysis pipeline that included sequence read alignment to the GRCh37/hg19 reference genome with the Burrows-Wheeler Aligner algorithm and Genome Analysis Toolkit variant calling. Subsequently, single-nucleotide variants or small insertion–deletions were annotated by a custom, in-house annotation pipeline. Copy number variants were assessed by the copy number inference from exome reads (CoNIFER) method. Variants in genes present in the *in silico* IEI panel (version 2.0.2) were filtered to retain coding, non-synonymous variants with population frequencies below 1% in our in-house database or population databases (GnomAD and dbSNP). Lastly, segregation analysis of the identified variants was performed in the daughter using standard Sanger sequencing.

### Cell culture

Human embryonic kidney 293 (HEK293T) cells were cultured at 37°C in Dulbecco’s modified Eagle’s medium (Life Technologies Laboratories, Grand Island, NY) with 10% fetal bovine serum. Peripheral blood mononuclear cells (PBMCs) from controls and patients were isolated by density gradient centrifugation with Lymphoprep (Progen Biotechnik, Heidelberg, Germany). PBMCs were washed twice with phosphate-buffered saline (Maastricht UMC, The Netherlands), resuspended at 1.0 × 10^7^ cells/mL in 1 mL Roswell Park Memorial Institute (RPMI) 1640 cell culture medium (Gibco, Thermo Fisher Scientific, Bleiswijk, The Netherlands) supplemented with 20% heat inactivated fetal calf serum (iFCS), and cryopreserved in 10% dimethylsulphoxide (DMSO) (Hybrid max, Sigma D2650) at −80 °C overnight before transfer to liquid nitrogen storage. For experiments, PBMCs were reconstituted and cultured in RPMI 1640 supplemented with Glutamax, 10% iFCS and 2% Penicillin/Streptomycin (Gibco, Thermo Fisher Scientific, Bleiswijk, The Netherlands). Stimulations were performed in 6-well plates overnight in the presence of 10% iFCS at 37°C and 5% CO_2_. Cells were then treated with 10 ng/mL tumor necrosis factor α (TNFα) (Gibco, Thermo Fisher Scientific, Bleiswijk, The Netherlands) for 2 hours, pelleted, and lysed in 100 uL lysis buffer. Supernatants were stored at − 20°C for immunoblot analysis.

### Plasmid construction and cell transfection

The pCMV3-neo-untagged-*TNFAIP3* wild-type plasmid, the mutated *TNFAIP3* plasmid, and the empty vector were purchased from Sino Biological Inc. (Beijing, China, HG12089-NY). HEK293T/17 cells (ATCC CRL-11268) were seeded in 6-well plates 24 hours before transfection. Empty vector, wild-type or mutant *TNFAIP3* plasmids were transfected using calcium phosphate. Forty-eight hours after transfection, cells were stimulated with 10 ng/mL TNFα for 2 hours, pelleted and lysed in 200 uL RIPA buffer (Thermo Fisher Scientific) with MS-Safe (Sigma). Lysates were stored at −20°C for immunoblot analysis.

### Immunoblot analysis

A20 expression in patient- and healthy-control-derived PBMCs was assessed by immunoblot. Protein lysates were separated on BIS-TRIS acrylamide gradient gels (6%–12%) and transferred to nitrocellulose membranes. Membranes were washed with Tris-buffered saline (TBS), blocked with TBS containing 3% bovine serum albumin and 0.1% Tween-20, then incubated overnight at 4°C with mouse monoclonal antibodies against A20 and β-actin (Santa Cruz). Immunoblots of transfected HEK293T cells were prepared similarly, using 12% gradient gels and blocking buffer containing 5% Blotto and 0.1% Tween-20. Membranes were probed with polyclonal antibodies against TRAF6 (Santa Cruz) and phospho-IκBα (Cell Signaling Technology). Detection was performed with horseradish peroxidase (HRP)-conjugated secondary antibodies (rabbit anti-mouse or goat anti-rabbit, as appropriate), and signal was visualized using the iBright Imager (Thermo Fisher). Band intensities were quantified with ImageQuant.

### Detection of circulating and in vitro induced cytokine production

For cytokine production measurements, isolated PBMCs were stimulated for 24 hours with LPS (10 ng/mL), phorbol 12-myristate 13-acetate (PMA, 12.5 ng/mL, Sigma-Aldrich), and ionomycin (500 ng/mL, Sigma-Aldrich), or Poly I:C (10 µg/mL) or left unstimulated in normal RPMI medium. In addition, cells were treated with the JAK inhibitor tofacitinib, p38 inhibitor or vehicle (0.1% DMSO). Concentrations of TNFα, IL-1β, IL-6, and IL-1Ra were measured in the culture supernatants using commercially available ELISA (Quantikine ELISA kits, R&D Systems) according to the manufacturer’s instructions. For all cytokines, measured values below the lower limit of detection are represented by this lowest detection value. Serum cytokines were measured using MILLIPLEX Multiplex Assays (Merck Millipore) using a FLexmap 3D system.

### Phosphoflow cytometry analysis

For phosphoflow cytometry experiments, PBMCs of patients and age-matched healthy controls (HCs) were cultured in U-bottom plates at a final concentration of 2 × 10^5^ cells in 200 μL per well containing culture medium supplemented with 5% FCS (Sigma-Aldrich) at 37°C and 5% CO_2_. Subsequently, cells were differentially stimulated with LPS (10 ng/mL), PMA (12.5 ng/mL, Sigma-Aldrich) and ionomycin (500 ng/mL, Sigma-Aldrich), TNFα (50 ng/mL) or Poly I:C (10 µg/mL) for 30 minutes. Next, cells were harvested and after surface staining of immune cell lineages (CD3, CD4, CD8, CD14, CD19, and CD56), the cells were fixed (Fixation Buffer, BD Biosciences) and permeabilized (Perm Buffer IV, BD Biosciences) and subsequently intracellularly stained for phosphorylated NF-κB (p65) (S529), STAT1 (Y701), STAT3 (Y705), mTOR (S2448), p70S6K (S235/236) and IκBα (S32/S36). Cells were analyzed on a 3-laser FC500 flow cytometer (Beckman coulter).

## Results

Based on the clinical phenotype with polyautoimmunity, immunodeficiency, and autoinflammatory features, diagnostic WES was performed to investigate the presence of an inborn error of immunity (IEI). This revealed a heterozygous NM_001270508.2: c.608T > G, p.(Leu203Arg) missense variant of *TNFAIP3* in both patients, as well as a heterozygous c.310T > C (p.Cys104Arg) variant of *TNFRSF13B* in the mother, a known risk factor that is present in 0.3%–0.5% of the normal population (GnomAD, v2.0.2).

**Figure 1:**
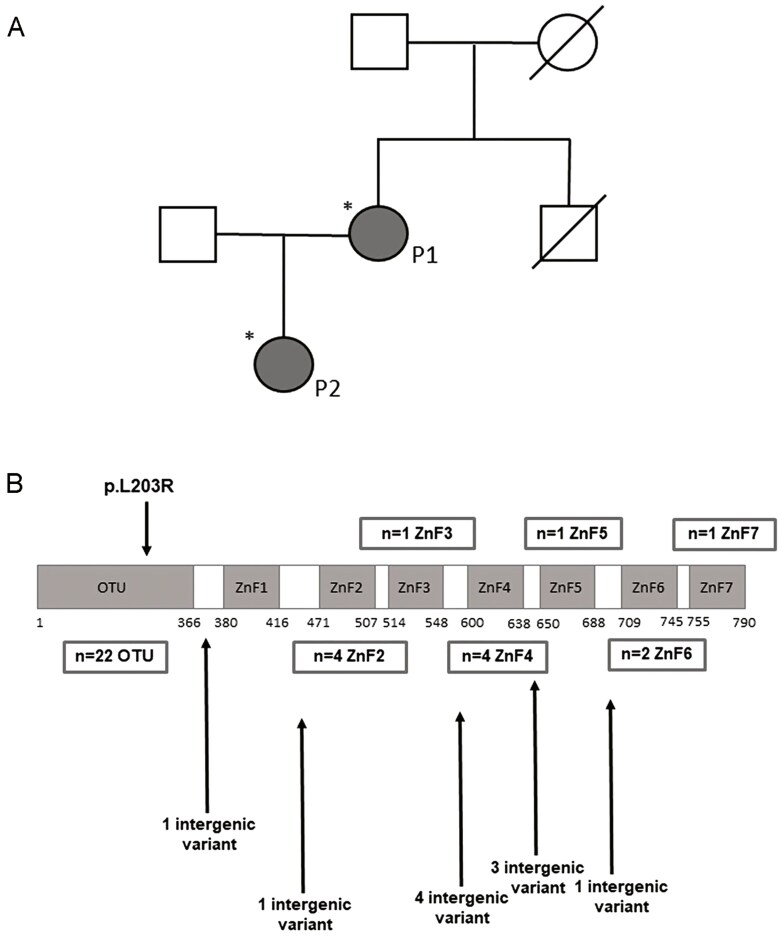
PEDIGREE of the family (A), P1 mother and P2 daughter. Brother of P1 passed away at the age of 40, had been residing abroad, and had a known medical history of rheumatism and thyroid disease. (B) The locations of the missense variants on the domain structure of the A20 protein. The panel includes all previously reported missense mutations and the variant evaluated in this study [8, 12].

The characteristics and pedigree of the mother and daughter with the novel *TNFAIP3* variant are summarized in Table 1 and Fig. 1A. The heterozygous c.608T > G (p.Leu203Arg) variant of *TNFAIP3* was not found in the gnomAD browser nor in any other population database. This variant is located in the OTU/deubiquitinylation domain (Fig. 1B). A computational analysis of the protein structure using 3D protein modeling (Protein Data Bank: 5LRX), UniProt annotations, and predictive software tools including MetaRNN [13] revealed that the p.Leu203Arg mutation introduces a larger, positively charged residue into the hydrophobic protein core. This alteration is located within the TRAF-binding region, where it may disrupt domain structure and function, consistent with a high MetaRNN score of 0.94, indicating a likely pathogenic variant effect.

**Table 1:** clinical characteristics of patient 1 (P1:mother) and 2 (P2:daughter)

	Age at onset	Autoantibodies	Igs (g/L)	Clinical presentation	Therapy
P1	18 years	Anti-TPO Anti-parietal cell Anti-GAD65 Anti-B2GP RF IgM	*IgA < 0.06* IgM 1.55 IgG 7.72	Orogenital ulcers Erosive RA DM type 1 Hashimoto thyroiditis Pernicious anemia Antiphospholipid syndrome	Methotrexate IgRT Cyclophosphamide Rituximab
P2	9 years	Anti-GAD65 DAT IgG	IgA 0.69 IgM 0.35 IgG 7.31 *IgG2 0.19*	Orogenital ulcers Recurrent sinusitis Folliculitis ITP Arthritis	Anakinra Adalimumab Infliximab Golimumab IgRT Rituximab

Abbreviations: anti-B2GP, anti-beta-2-glycoproteins; anti-GAD65, anti-glutamic acid decarboxylase 65 antibodies; DAT, direct antiglobulin testing; DM, diabetes mellitus; IgRT, immunoglobulin replacement therapy; ITP, immune thrombocytopenia; RA, rheumatoid arthritis; RF, rheumatoid factor; TPO, thyroid peroxidase.

### Functional characterization of the novel TNFAIP3 variant

#### Reduced A20 expression in patient PBMCs and HEK293T expression model

**Figure 2:**
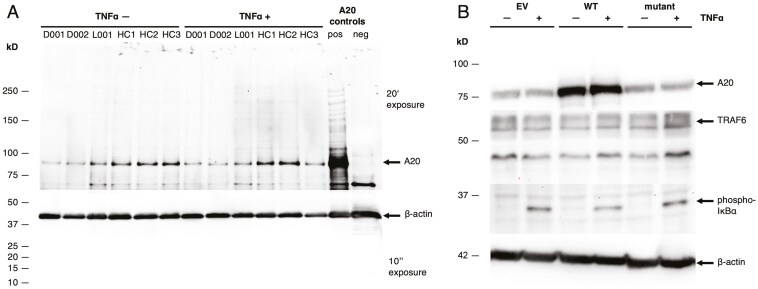
reduced A20 expression in patient-derived PBMCs and in a mutant overexpression model. (A) Immunoblot analyses of A20 and β-actin in PBMCs before and after TNFα stimulation. The expression levels of A20 in D001 (patient 1) and D002 (patient 2) were reduced compared to HCs and L001 (CVID patient control with *TNFAIP3* LoF mutation c.1309del (p.Ala437Profs)). A20 positive control: PBMC lysate with high A20 expression. A20 negative control: granulocyte lysate. Exposure times: 20 minutes for A20, 10 seconds for β-actin. (B) Immunoblot of HEK293T cells transfected with wild-type (WT) or mutant (Mut) A20 constructs, or with empty vector (EV), with and without TNFα stimulation. Cells expressing the mutant construct show reduced A20 levels and increased expression of TRAF6 and phosphorylated IκBα (phospho-IκBα), indicating enhanced NF-κB pathway activation. β-actin was used as a loading control. Exposure times: 30 seconds for A20, 3 seconds for β-actin, 120 seconds for TRAF6 and 900 seconds for phosphorylated IκBα.

To investigate A20 expression in patients and HCs, immunoblot analysis was performed on PBMCs, which showed reduced A20 expression in patient-derived PBMCs, both before and after TNFα stimulation (Fig. 2A). Quantification of band intensities for A20 and actin, showed a significantly lower A20/actin ratio in patients compared to HCs, particularly after stimulation with TNFα (P1: 0.205; P2 0.218; L001 0.453 versus mean ratio HC:0.802, see Supplementary Table 1). To assess the functional impact of the missense variant, we used an ectopic expression model in HEK293T cells transfected with either wild-type or mutant constructs. In this model, mutant A20 expression was consistently reduced, while levels of TRAF6 and, to a lesser extent, phosphorylated IκBα were increased following TNFα stimulation (Fig. 2B). After stimulation, the A20/actin ratio was 1.18 for wild-type and 0.28 for mutant. Corresponding values for TRAF6 were 0.57 (wild-type) and 0.92 (mutant), and for phosphorylated IκBα were 0.13 (wild-type) and 0.18 (mutant).

### *Increased NF-κB pathway activation in CD8+, CD4+*, *and CD14+ cells from mutant A20 patients*

**Figure 3:**
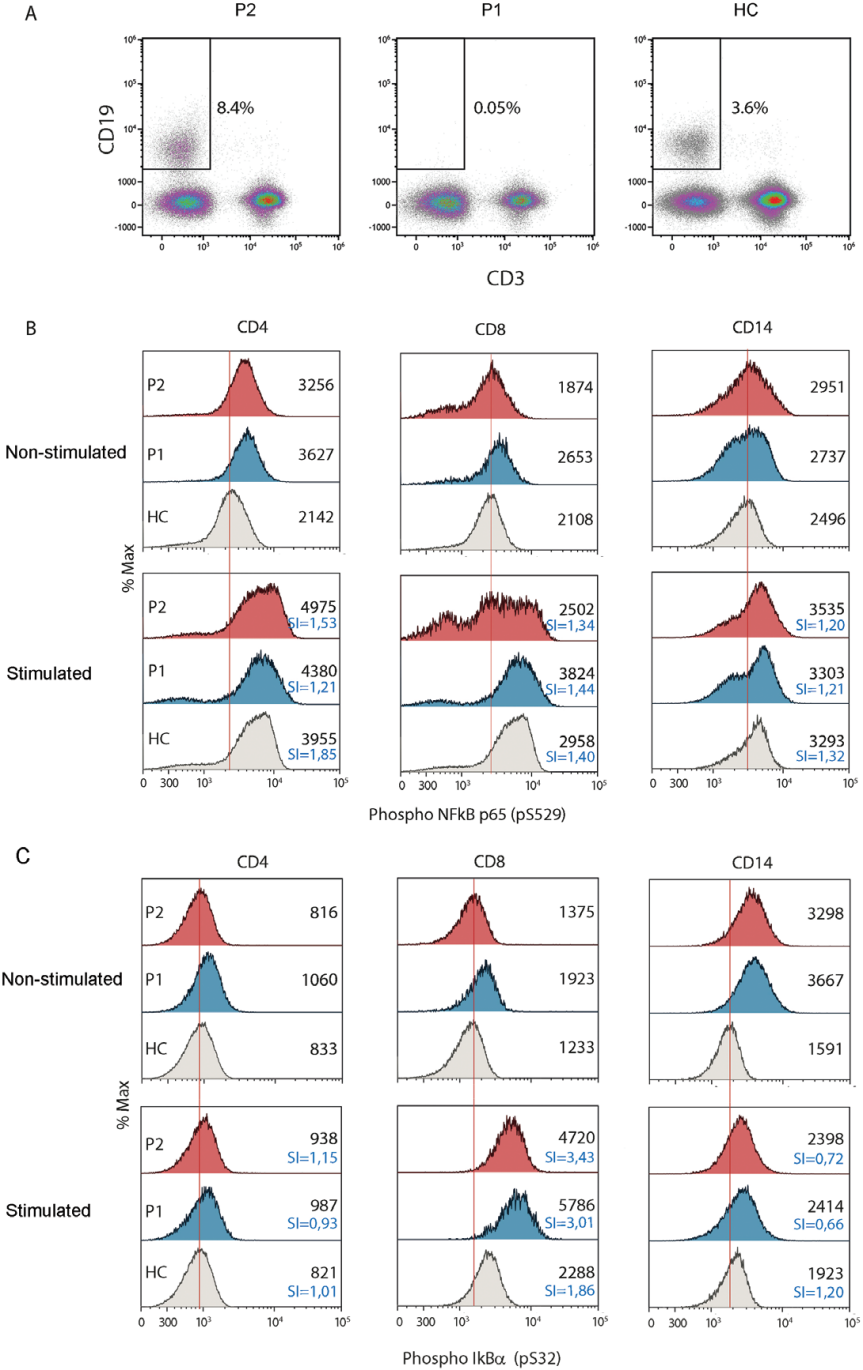
increased phosphorylation of NF-κB and IκBα in patient T cells. Flow cytometry analysis showed depletion of CD19 + B cells in P1 (A). Phosphoflow cytometry of p65 NF-κB (B) and IκBα (C) in CD4+, CD8 + and CD14 + gated cells (indicated above histograms) from HA20 patients 1 (P1, mother) and 2 (P2, daughter) and healthy control (HC). The mean fluorescence intensity (MFI) is depicted in each histogram. SI, stimulation index.

Given that A20 is a critical negative regulator of NF-κB signaling, our research aimed to elucidate whether reduced levels of A20 were linked to a dysregulation in NF-κB activation. This involved analyzing the phosphorylation status of the p65 and IκBα NF-κB subunits in both unstimulated and P/I stimulated PBMCs from patients. Our analysis focused first on CD3+ T cells (CD4+ and CD8+) and monocytes (CD14+), considering the depletion of B cells observed in patient 1 following treatment with rituximab (see Fig. 3A). In the constitutive state, our findings revealed higher levels of p65 NF-κB phosphorylation in CD4+ and CD14+ cells from patients compared to the HC, while no significant disparity was observed in CD8+ cells (see Figure 3B). For phospho-IκBα, elevated levels in the constitutive state are most pronounced in CD14+ cells (Fig. 3C). The same result was observed in the levels of p38 MAPK (Supplementary Fig. 1). Upon P/I stimulation, CD8+ cells from patients demonstrate increased IκBα NF-kB phosphorylation compared to HC, whereas this was not observed in CD4 + and CD14 + cells (Fig. 3B and C).

### Increased IκBα phosphorylation in stimulated CD19+ cells from mutant A20 patients

**Figure 4:**
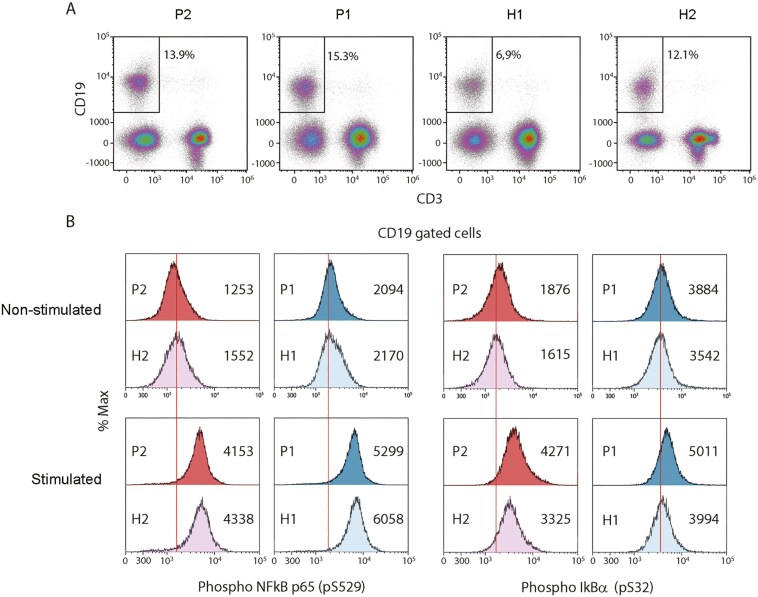
increased phosphorylation of IκBα in stimulated patient B cells. Flow cytometry analysis (A) showed the constitution of CD19 + B cells in P1. Phosphoflow cytometry of CD19 + B cells (indicated above histograms) of HA20 patient 1 (P1, mother) and 2 (P2, daughter) and age-matched healthy controls (H1, H2). The mean fluorescence intensity (MFI) is depicted in each histogram. SI: stimulation index.

In the subsequent phase of our study, conducted 1 year later, B cells were successfully reconstituted in patient 1 (see Fig. 4A), while at that time patient 2 underwent treatment with TNF inhibitors. Phosphoflow cytometry was once again conducted on freshly isolated PBMCs from both patients to assess NF-κB pathway activation in both unstimulated and P/I stimulated PBMCs, now with a focus on B cells. In the constitutive state, no differences were observed in the phosphorylation of p65 NF-κB and IκBα between patients and HCs. Following P/I stimulation, an increased phosphorylation of IκBα was observed in the B cells, whereas no differences were observed in the levels of p65 NF-κB phosphorylation compared to HCs (Fig. 4B).

### Pro-inflammatory cytokine profiles in mutant A20 patients

**Figure 5:**
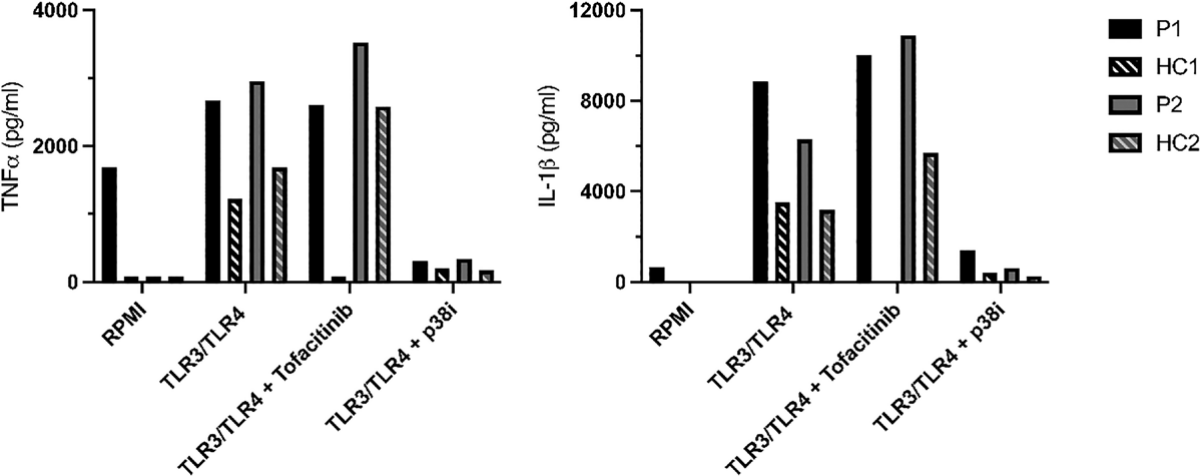
concentration of innate cytokines in culture supernatants of *in vitro* stimulated peripheral blood mononuclear cells. Isolated PBMCs were stimulated for 24 hours with poly I:C (10 μg/mL) and LPS (10 ng/mL) or left unstimulated in normal RPMI medium. In addition, cells were treated with the JAK inhibitor tofacitinib, p38 inhibitor or vehicle (0.1% DMSO). Concentrations of TNFα (A) and IL-1β (B) were measured in the culture supernatants using ELISA.

**Figure 6:**
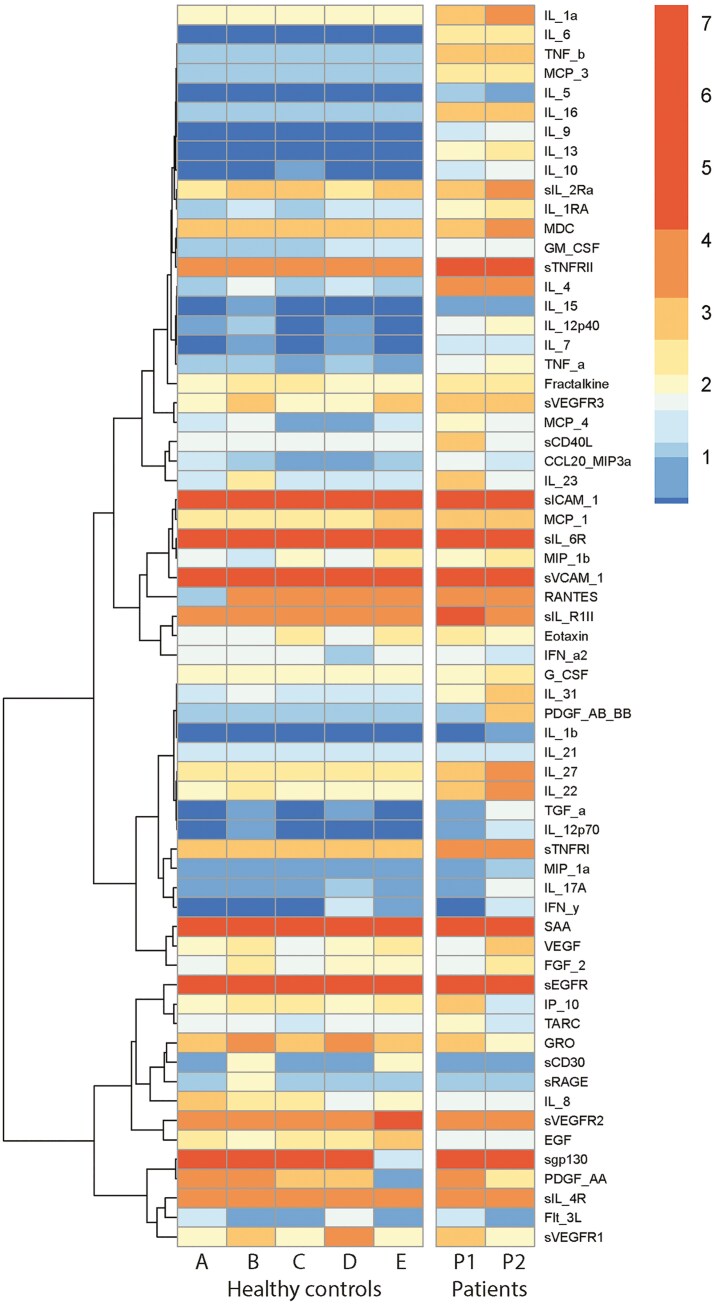
increased pro-inflammatory cytokines in patient serum. Serum profiling using multiplex Luminex technology of HA20 patients 1 (P1, mother) and 2 (P2, daughter) versus healthy controls. Log10-transformed data are shown.

Since p65 is involved in the upregulation of inflammatory mediators [1], we assessed circulating pro-inflammatory cytokines and induced cytokine production *in vitro*. At the time of measurement, both patients were in stable remission: the mother following rituximab treatment but with B-cell reconstitution, and the daughter currently receiving TNF blockers. Downstream cytokine expression upon *in vitro* stimulation of PBMCs after induction of TLR3/TLR4 signaling by LPS and Poly I:C for 24 hours showed elevated concentrations of TNFα and IL-1β in comparison to healthy individuals, which were abrogated after p38 inhibition and not after tofacitinib, a selective JAK1/3 inhibitor (Fig. 5). Moreover, higher basal production of IL-6 and IL-1Ra were observed in P1 (Supplementary Fig. 2). Measurement of circulating serum cytokines demonstrated a clear pro-inflammatory profile in both patients as compared to the HCs, including markedly elevated levels of IL-1α, IL-6, TNFβ, MCP3, and to a lesser extent IL-4, IL-13, IL-16, VEGFR3, and sTNFR2 (IL22/IL27) (Fig. 6).

### Increased phosphorylation of mTOR, p70S6K, and STAT1

**Figure 7:**
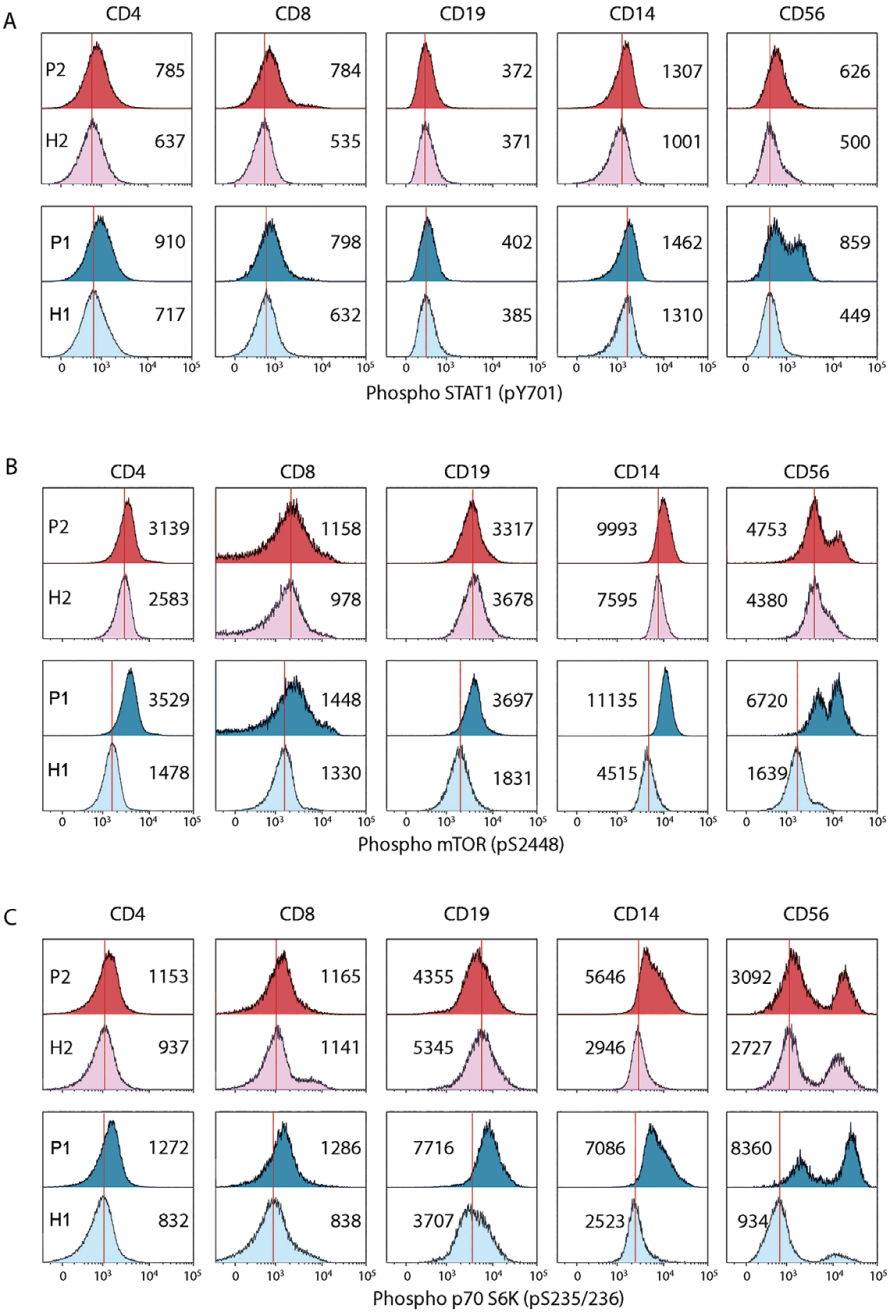
increased baseline phosphorylation of STAT1, mTOR, and p70S6K in patients. Phosphoflow cytometry of (A) STAT1, (B) mTOR and (C) p70S6K in CD4+ and CD8+ T cells, CD19+ B cells, CD14+ monocytes and CD56+ NK cells (indicated above histograms) of HA20 patients 1 (P1, mother) and 2 (P2, daughter) and age-matched healthy controls (H1, H2). The mean fluorescence intensity (MFI) is depicted in each histogram. P, patient; H, healthy control.

Other studies have also indicated overactivation of the IFNy pathway in addition to the NF-κB pathway in HA20 [5, 9, 10]. Our research findings, which indicated that a JAK1/3 inhibitor did not lead to a reduction in the inflammatory cytokines TNFα and IL-1β, prompted us to investigate whether we could also observe this hyperactivation of the IFNy pathway in our patients or if the mTOR pathway played a more significant role. PBMCs from patients and their age-matched HCs were either left unstimulated or were *ex vivo* stimulated with P/I or Poly IC/LPS, followed by an evaluation of STAT1 and STAT3 phosphorylation. In the constitutive state, we observed significantly higher levels of phosphorylated STAT1 in the patients, especially in T cells, monocytes and NK cells, when compared to HCs (Fig. 7A). This contrasts with to the levels of phosphorylated STAT3, which were not elevated in the patients. Following stimulation, we did not observe any differences in STAT1 and STAT3 phosphorylation between patients and the HCs (Supplementary Fig. 3A and B). Increased phosphorylation of mTOR was primarily detected in CD4+ T cells, CD14+, and CD56+ cells in the constitutive, unstimulated PBMCs (Fig. 7B). This elevation in phosphorylation was also evident in the mTORC1 substrate S6K in unstimulated cells (Fig. 7C), while no such pattern was observed in substrate 4E-BP1. Upon stimulation, no differences were noted between patients and HCs (Supplementary Fig. 4).

## Discussion

In the current study, we have described the clinical and functional phenotype of a novel *TNFAIP3* variant in a family with a predominant autoimmune phenotype. Initially, HA20 was described as a typical autoinflammatory disease [1], but it is now known that one-third of patients also exhibit autoimmunity, such as thyroiditis, type 1 diabetes, and SLE [8]. In the family described here, the clinical presentation is primarily characterized by autoimmune manifestations, including the previously reported thyroiditis, pernicious anemia and ITP. However, it is the first description of erosive RF + RA and secondary APS in a HA20 patient. The link between HA20 and autoimmune disorders is established [5], yet the exact mechanisms underlying its role in the development of autoimmunity remain unclear. We demonstrated that in addition to the reduced negative feedback of the NF-κB signaling pathway, there is a clear activation of STAT1 (but not STAT3) and the mTOR pathway. This suggests the involvement of A20 in derailment of the mTOR pathway, thereby providing a novel insight into the emergence of autoimmunity in HA20.

A20 inhibits NF-κB signaling via two distinct mechanisms: deubiquitination, facilitated by its N-terminal OTU domain, and ubiquitin ligase activity, executed by its C-terminal zinc finger domains. Although the OTU and zinc finger domains of A20 possess diverse biochemical roles and target various proteins and pathways, their effects ultimately converge to suppress NF-κB activity [14]. An increase of cytokines production, as well as of phosphorylation levels of NF-kB has been described in patients carrying mutations in both OTU and Zn finger domain [1, 7]. The heterozygous c.608T > G (p.Leu203Arg) variant in *TNFAIP3* found in both patients presented here affects the OTU domain and represents a novel variant, previously unreported and not present in major gene variant databases. We demonstrated that this mutation results in an altered protein with reduced A20 expression in both patient-derived cells and an ectopic expression model. This reduction was accompanied by activation of the NF-κB pathway, as shown by immunoblot analysis, supporting a pathogenic role for the variant. Additionally, phosphoprotein analysis revealed elevated NF-κB phosphorylation in unstimulated monocytes and CD4^+^ cells, as well as in CD8^+^ and B cells following stimulation. The increase in phosphorylation of IκBα can also possibly be due to reduced IκBα protein degradation, potentially through A20. Furthermore, it was related to pro-inflammatory cytokine dysregulation displaying increased production of the pro-inflammatory cytokines TNFα and IL-1β, which were abrogated after p38 inhibition. Persistent or abnormal NF-κB activation can disrupt self-tolerance, promoting the survival and activation of autoreactive B cells [15]. These cells may become plasma cells, secreting autoantibodies. Additionally, a pro-inflammatory environment further stimulates these B cells and T cells, increasing autoantibody production. Indeed, findings in mice models demonstrate that A20 plays a crucial role in the development of autoantibodies and functions of B cells [16].

In addition to diminished regulation of the NF-κB pathway, previous research has demonstrated hyperactivation of the IFNγ pathway in HA20 patients [5, 9, 10]. This includes significantly elevated levels of IFNγ-inducible chemokines CXCL9 and CXCL10, as well as higher phosphorylated STAT1 levels in HA20 patient monocytes compared to healthy individuals upon IFNγ stimulation [5]. Besides STAT1, increased phosphorylation of STAT3 was observed. Additionally, the successful use of JAK inhibitors further underscores A20’s role in modulating IFN signaling [9, 10]. However, we did not detect elevated IP-10/CXCL10 or IFNγ levels in patient sera, nor did we observed increased levels of STAT3 phosphorylation. This was previously confirmed by de Wilde et al. in a myeloid-specific A20-deficient mice model, where STAT1 but not STAT3 expression was enhanced leading to STAT1-dependent joint inflammation [17]. Our findings regarding STAT1 phosphorylation align with prior studies, affirming STAT1’s role in HA20. However, after the addition of tofacitinib in the *in vitro* induced cytokine assays, the production of the inflammatory cytokines TNFα and IL-1β remained unaffected. This observation hold therapeutic consequences for our patients, leading us to investigate the interplay between A20 and the mTOR pathway, rather than the STAT1 pathway. Both the mTOR and STAT pathways are pivotal in regulating the development, survival, and functionality of various immune cells, including CD8+ cytotoxic T cells, CD4+ helper T cells, regulatory T cells, dendritic cells, and monocytes. These pathways are integral to both innate and adaptive immune responses, and their dysregulation is associated with autoimmune conditions [18].

Our results clearly show a relation that mutA20 affects mTOR signaling, which was reflected by an enhanced phosphorylation status of both mTOR and S6K. The mTOR protein kinase forms two structurally and functionally distinct complexes called mTOR Complex 1 (mTORC1) and mTOR Complex 2 (mTORC2). They are comprised of different proteins but both include mTOR-interacting protein (DEPTOR), an inhibitor of mTOR signaling. After activation, mTORC1 stimulates mRNA translation by phosphorylating downstream effectors such as p70S6 kinase (S6K) and eukaryotic initiation factor 4E-binding protein 1 (4E-BP1). Studies reveal mTOR’s involvement in various body signaling pathways, like phosphoinositide 3-kinase (PI3K)/AKT [19]. TNFAIP3 directly impacts the mTOR signaling cascade by inhibiting the ubiquitination and subsequent activity of the mTOR complex, thereby promoting increased autophagy in NK and CD4+ T cells [20, 21]. Moreover, a direct inhibitory effect of A20 on the mTORC2/Akt/Rac1 signaling axis was found in hepatocellular carcinoma cells, due to the direct interaction between A20 and mTORC2 complex [22]. Furthermore, in human ankylosing spondylitis, A20 induces early autophagy by stabilizing DEPTOR and attenuates the symptoms of the disease [23]. Autophagy is crucial in autoimmunity, regulating inflammation, antigen presentation, lymphocyte homeostasis, and the removal of self-antigens, thus maintaining immune balance. Dysregulated autophagy can lead to excessive inflammation and the accumulation of self-reactive cells, contributing to autoimmune disease development. Limited research exists on the regulation of autophagy by A20. Vetters *et al.* revealed that rapamycin, a recognized mTOR inhibitor, strongly protected NK-A20^−/−^ cells from death [20], providing new insights in the treatment of patients with A20 haploinsufficiency. The potential utilization of mTOR inhibitors like everolimus or sirolimus/rapamycin warrants consideration in therapeutic strategies.

There are limitations to our study. First, the analysis of PBMCs might have been suboptimal because of immunosuppressive treatment in the patients. This includes the absence of B cells in the mother during the initial analysis as a result of rituximab treatment, and the potentially altered lymphocyte response of the daughter during the second experiment, due to treatment with TNF blockade. Additionally, both patients were in clinical remission at the time of the cytokine assays, the mother following rituximab treatment with evidence of B-cell reconstitution, and the daughter while receiving TNFα inhibition. As a result, it is challenging to establish a correlation between cytokine responses, treatment effects, and clinical outcomes. Secondly, although we did not directly assess transcriptional activity, the reduced abundance of the mutant protein, together with increased downstream signaling, suggests that the variant may affect protein stability. Finally, the mother possesses a variant in the *TNFRSF13B* gene, which encodes the transmembrane activator and calcium-modulating cyclophilin ligand interactor (TACI), a receptor crucial for B-cell function and immunoglobulin production and associated with common variable immunodeficiency (CVID) [24]. TACI variants have been identified as genetic risk factors associated with lymphoproliferation and autoimmune manifestations. These risk variants occur at relatively high allele frequencies in the general population, indicating substantial variability in clinical expression and penetrance. Functional studies demonstrate that *TNFRSF13B* variants impair TACI signaling by disrupting ligand binding, receptor oligomerization, and downstream MYD88 signaling, thereby affecting B cell homeostasis, isotype switching, and antibody responses [25]. The increased prevalence of the p.(Cys104Arg) variant in patients with primary antibody deficiency was recently confirmed by our study group [26], supporting a causal relationship. Although this variant may contribute to the development of IgA deficiency and autoimmunity in the mother, its absence in the daughter suggests that it is not a critical modifier of the clinical phenotype. To our knowledge, no previous data have been published on the interaction of the *TNFRSF13B* and *TNFAIP3* variant. Nevertheless, it may be hypothesized that the *TNFRSF13B* variant acts as a genetic modifier, enhancing autoimmunity, which appears more pronounced in the mother.

In conclusion, we have identified a novel pathogenic variant in *TNFAIP3* associated with HA20, confirming its functional impact. Our findings reveal that HA20 not only triggers enhanced NF-κB activation but also activates the mTOR pathway, potentially through the interaction with STAT1 but not STAT3. These alterations disrupt immune tolerance, highlighting a complex interplay in autoimmunity development. The spectrum of HA20 manifestations is expanding, with our findings suggesting that autoimmune manifestations may predominate over traditional autoinflammatory Behçet-like symptoms in certain patients. This observation opens the door to alternative immunosuppressive therapies, such as JAK1/2 or mTOR inhibitors.

## Supplementary Material

uxaf048_suppl_Supplementary_Materials_1

## Data Availability

Almost all data are incorporated into the article and its Online Supplementary Material. Additional data is available on request.
