# Supplementary material for: A novel missense variant in TNFAIP3 associated with autoimmunity reveals the contribution of STAT1/mTOR pathways
Source: Clin Exp Immunol. 2025 Jul 25;219(1):uxaf048. doi: 10.1093/cei/uxaf048 (PMC12457938; doi:10.1093/cei/uxaf048)

**Supplementary tables and figures**

**Suppl Table 1. Locally corrected volume of ratio A20/actin through quantification of band intensities, before and after stimulation with TNFα. P1:D001; P2:D002. L001: CVID patient control with *TNFAIP3* LoF mutation c.1309del (p.Ala437Profs). HC: healthy control.**

|  | **Volume**  no TNFα | **Density**  no TNFα | **Local corr volume**  no TNFα | **Volume**  After TNFα | **Density**  After TNFα | **Local corr volume**  After TNFα |
| --- | --- | --- | --- | --- | --- | --- |
| **D001** | 0.533 | 0.480 | 0.155 | 0.465 | 0.470 | 0.205 |
| **D002** | 1.101 | 0.982 | 0.434 | 0.488 | 0.461 | 0.218 |
| **L001** | 1.005 | 1.129 | 0.510 | 0.892 | 0.979 | 0.453 |
| **HC 1** | 1.037 | 1.023 | 0.715 | 1.354 | 1.707 | 1.040 |
| **HC 2** | 1.114 | 1.316 | 0.835 | 1.632 | 1.551 | 1.150 |
| **HC 3** | 1.187 | 1.393 | 1.037 | 1.346 | 1.745 | 0.216 |

**Suppl Figure 1. Increased phosphorylation of p38 MAPK in patient T-cells and monocytes in the constitutive state. Phosphoflow cytometry of p38 MAPK in CD4+, CD8+ and CD14+ gated cells (indicated above histograms) from HA20 patient 1 (P1, mother) and 2 (P2, daughter) and age matched healthy control (HC) in the constitutive state. The mean fluorescence intensity (MFI) is depicted in each histogram.**

**
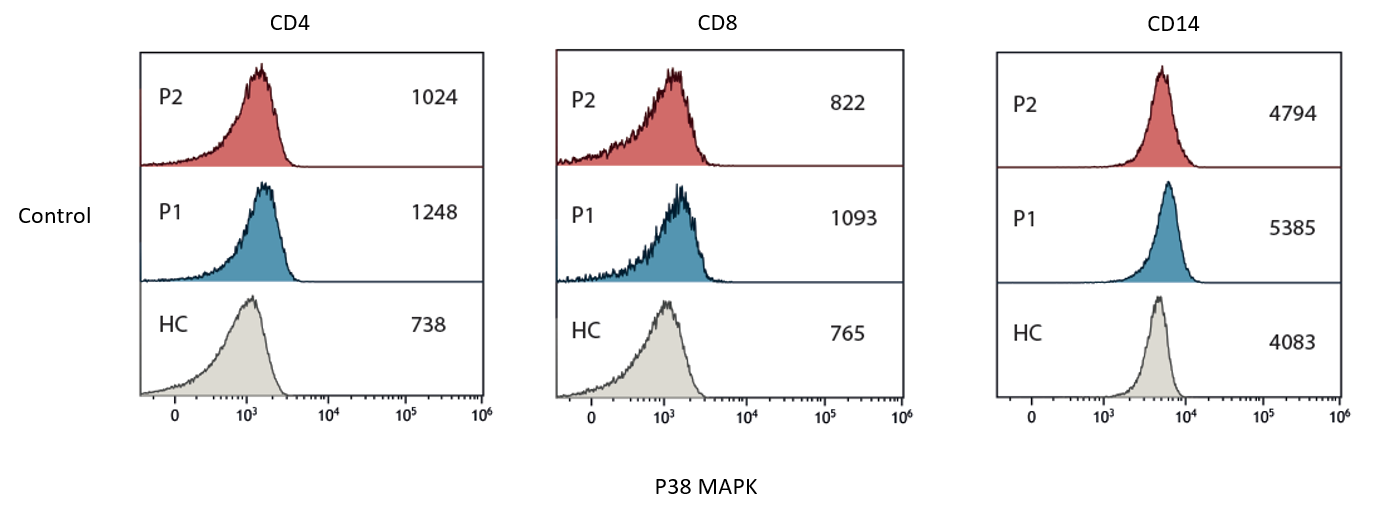
**

**Suppl Figure 2. Concentration of innate cytokines in culture supernatants of *in vitro* stimulated peripheral blood mononuclear cells. Isolated PBMCs were stimulated for 24h with poly I:C (10 μg/mL) and LPS (10 ng/ml) or left unstimulated in normal RPMI medium. In addition, cells were treated with the JAK inhibitor tofacitinib, p38 inhibitor or vehicle (0.1% DMSO). Concentrations of IL-6 and IL-1RA were measured in the culture supernatants using ELISA.**


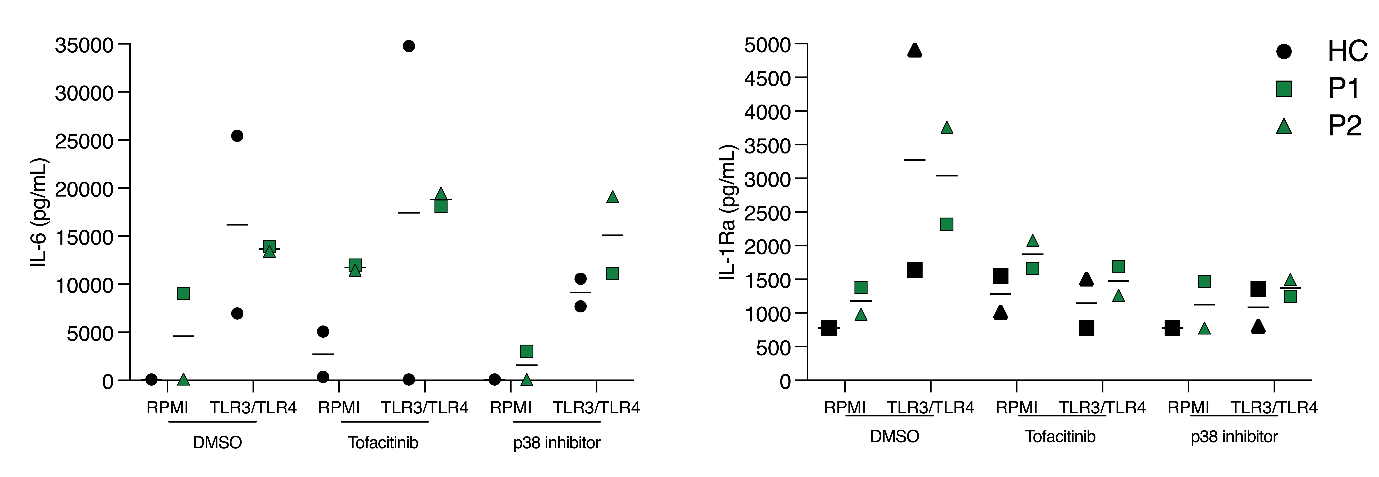


**Suppl figure 3. Following P/I stimulation, no difference in phosphorylation of STAT1 and STAT3 was observed between patients and HCs. Phosphoflow cytometry of (A) STAT1, (B) STAT3 in CD4+ and CD8+ T-cells, CD19+ B-cells, CD14+ monocytes and CD56+ NK cells (indicated above histograms) of HA20 patient 1 (P1, mother) and 2 (P2, daughter) and age matched healthy controls (H1, H2). P: patient. H: healthy control.**

**
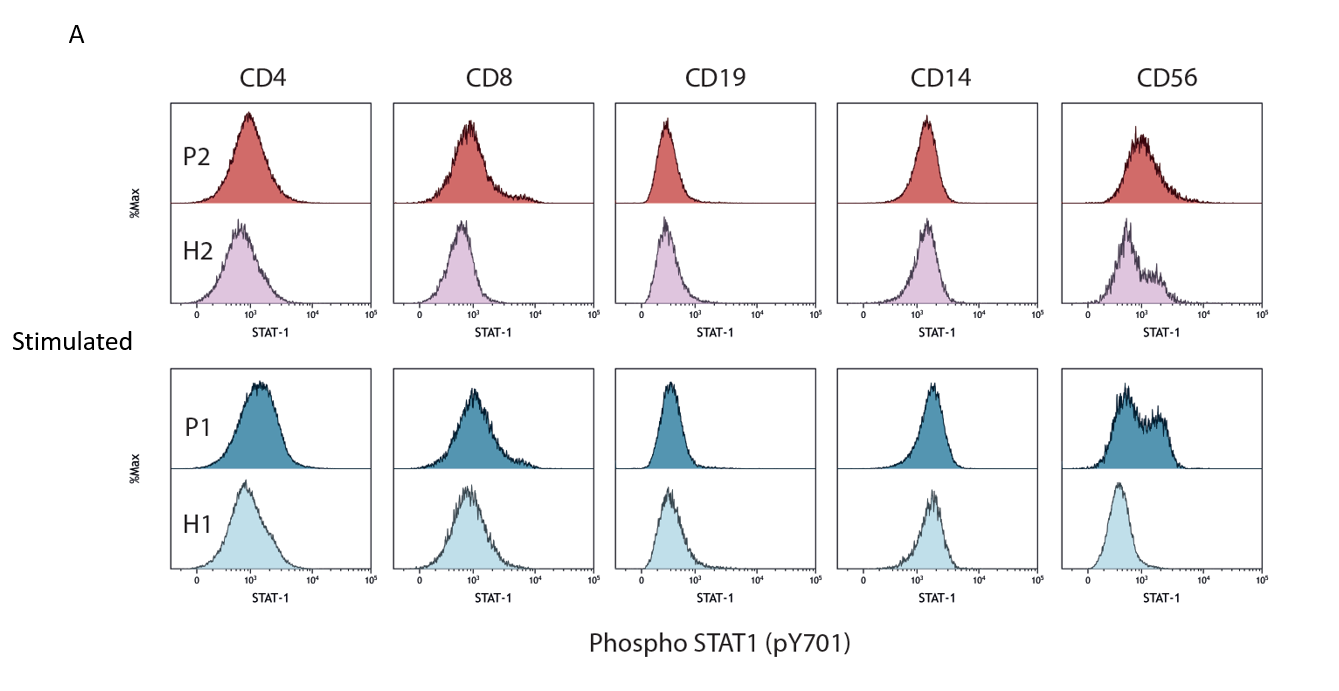
**


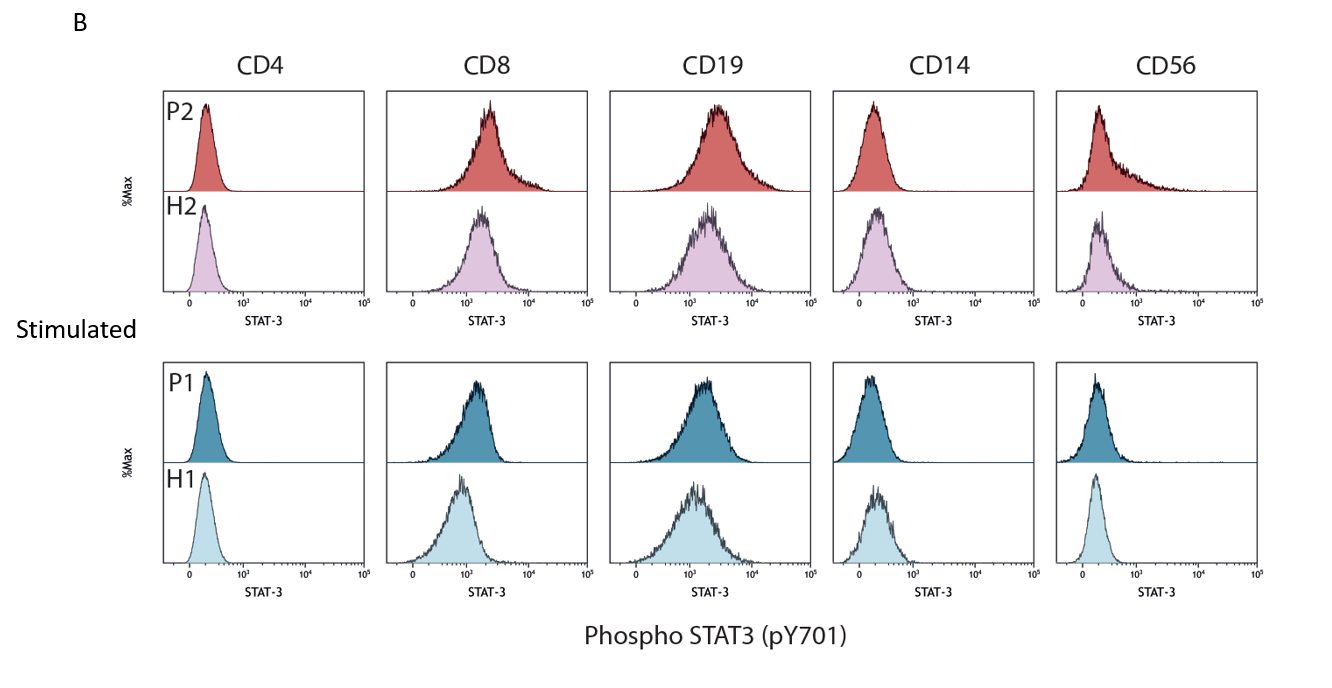


**Suppl figure 4.** **Following P/I stimulation, no difference in phosphorylation of mTOR and S6K was observed between patients and HCs. Phosphoflow cytometry of (A) mTOR, (B) S6K in CD4+ and CD8+ T-cells, CD19+ B-cells, CD14+ monocytes and CD56+ NK cells (indicated above histograms) of HA20 patient 1 (P1, mother) and 2 (P2, daughter) and age matched healthy controls (H1, H2). P: patient. H: healthy control.**


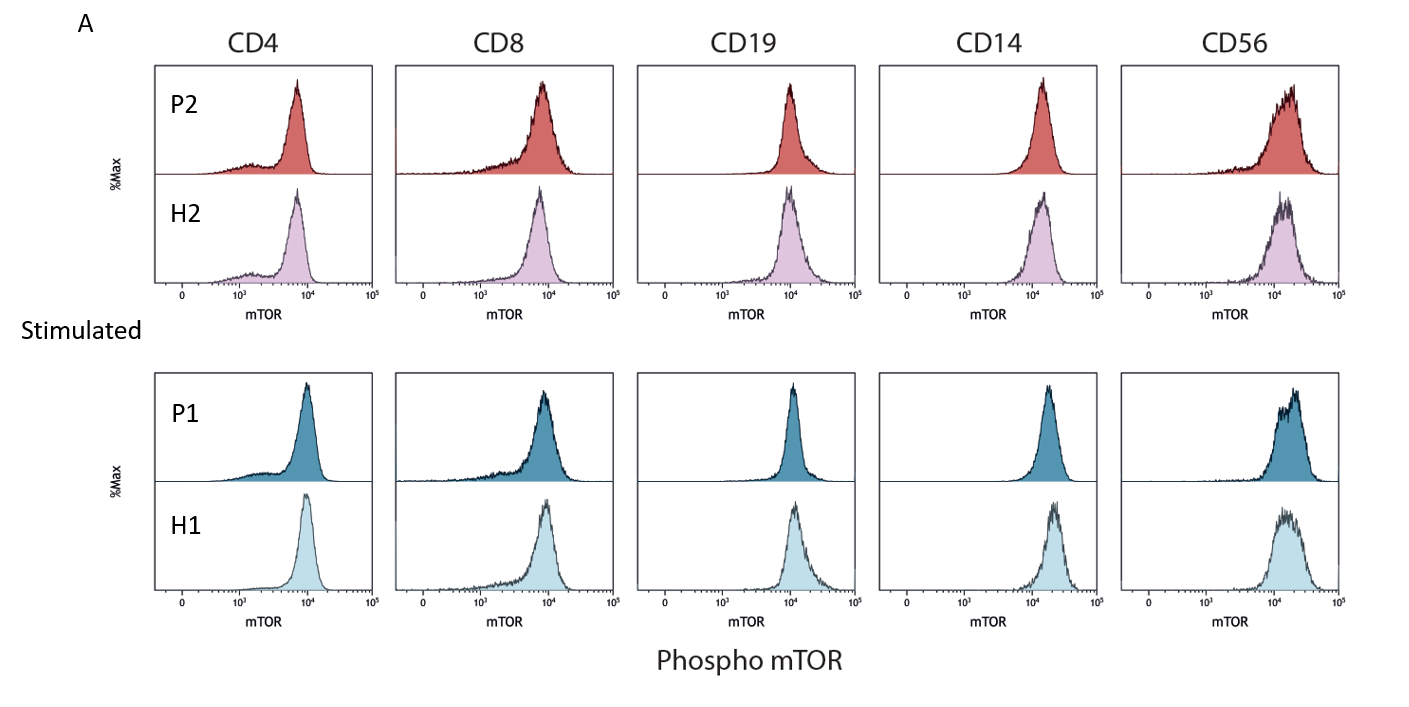


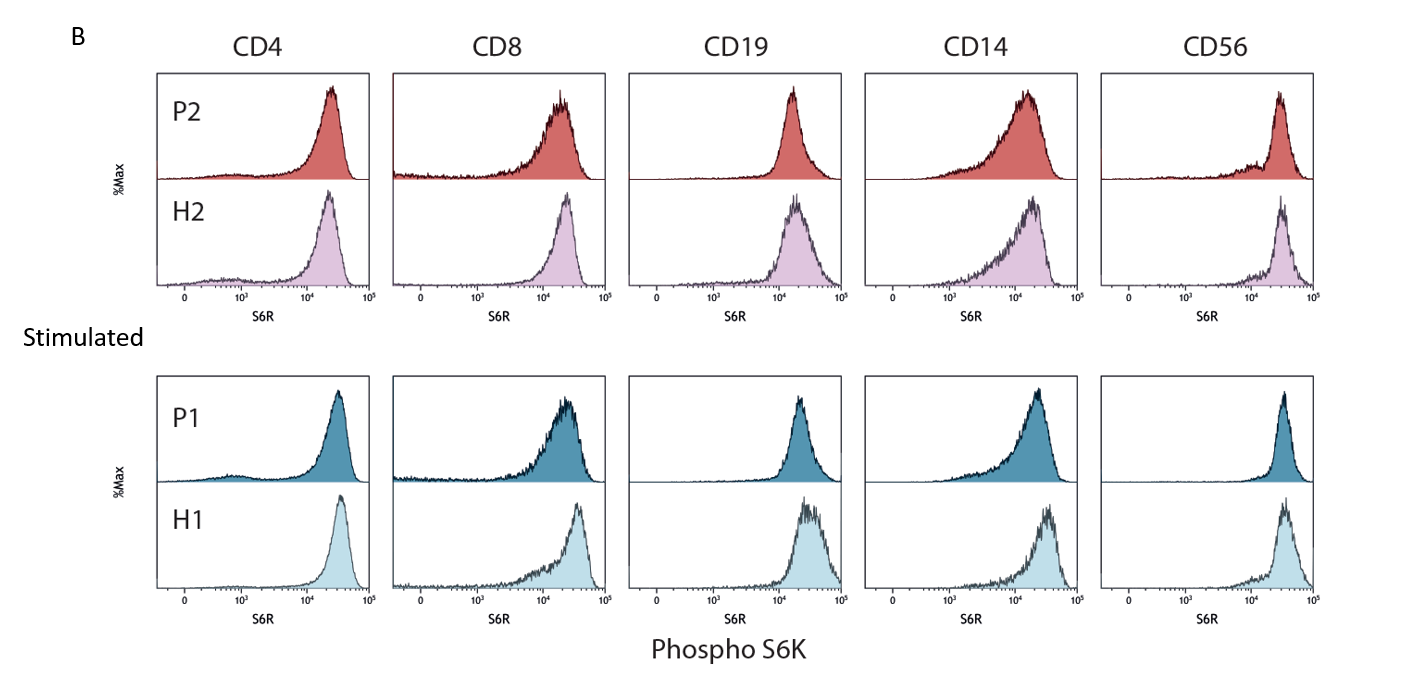

Supplement: uxaf048_suppl_Supplementary_Materials_1 [file uxaf048_suppl_supplementary_materials_1.docx]
